# Complete Chloroplast Genome Sequence of *Chenopodium album* from Northeastern India

**DOI:** 10.1128/genomeA.01150-17

**Published:** 2017-11-22

**Authors:** Rajkumari Jashmi Devi, Biseshwori Thongam

**Affiliations:** Plant Systematics and Conservation Laboratory, Institute of Bioresources and Sustainable Development (IBSD), Takyelpat, Imphal, Manipur, India

## Abstract

*Chenopodium album* belongs to the complex genus *Chenopodium* of the family Amaranthaceae. It is an economically and medicinally important plant. We report here the first complete chloroplast genome sequence of *C. album* from northeastern India. This study shall add extensive information on the evolutionary relationships of the genus *Chenopodium*.

## GENOME ANNOUNCEMENT

The genus *Chenopodium* of the family Amaranthaceae comprises about 150 herbaceous, suffrutescent, and arborescent perennial species (1). Many of the species belonging to this complex genus are not easily defined due to a lack of distinctive macroscopic morphological characteristics (2–4). *Chenopodium album* Linn. is one of the economically important plants of this genus. It is reported as a wild, edible, medicinal plant from Manipur, northeastern India (5). A *C. album* cultivar from the Indian Himalayan region having white-colored seed is morphologically similar to *Chenopodium quinoa*. This raised a question on the taxonomic status of *C. album* (6). A lack of solid knowledge about the *Chenopodium* species led to the need for molecular characterization for proper identification. This study reports the first complete chloroplast genome sequence of *Chenopodium album* from northeastern India.

Plant material of *C. album* was collected from Purul (25°22′30.14″N, 94°13′43.05″E) in the Senapati district of Manipur, India, and was grown in the greenhouse of the Institute of Bioresources and Sustainable Development (IBSD), Imphal, India. Chloroplast DNA for whole-plastid-genome sequencing was isolated from fresh leaves according to the modified high-salt method (7). The complete chloroplast genome sequence was determined from the high-quality 2 × 250-bp paired-end library using the Illumina HiSeq 2500 platform. The adapter sequences and low-quality bases (Q20) were trimmed from raw reads using AdapterRemoval version 2.2.0 (8). The complete chloroplast genome sequence was then assembled with the Mira version 4.9.6 software (9). The protein-, rRNA-, and tRNA-coding genes were annotated using DOGMA, CPGAVAS, and tRNAscan-SE 2.0, respectively (10–12). Manual editing was also carried out by comparison with the chloroplast genome sequence of *Chenopodium* species deposited in NCBI GenBank. The circular map of the genome was drawn using OGDraw version 1.2 (13).

The complete chloroplast genome sequence of *C. album* is 150,272 bp in length. It has a quadripartite structure, with a large single copy (LSC) of 82,417 bp, small single copy (SSC) of 23,271 bp, and two inverted repeats of 22,292 bp (IRA and IRB). The genome has an overall GC content of 36.97%. It has a total of 113 genes comprising 80 protein-coding genes, 29 tRNA-coding genes, and 4 rRNA-coding genes. The two inverted repeats have 17 duplicated genes, *viz*. *rrn*5, *rrn4.5*, *rrn23*, *trnA*-UGU, *trnI*-GAU, *trnV*-GAC, *trnL*-CAA, *trnI*-CAU, *rpl2*, *rpl23*, *rps*7, *rps12*, *ycf2*, *ycf15*, *ycf68*, and *ndhB*. This study shall add extensive information on the genetics and evolutionary relationship analysis of the genus *Chenopodium*.

### Accession number(s).

The complete chloroplast genome sequence of *C. album* is submitted to NCBI GenBank under the accession number MF418659.
